# Impact of Stress on Gamma Oscillations in the Rat Nucleus Accumbens During Spontaneous Social Interaction

**DOI:** 10.3389/fnbeh.2019.00151

**Published:** 2019-07-10

**Authors:** Ann Mary Iturra-Mena, Marcelo Aguilar-Rivera, Marcia Arriagada-Solimano, Catherine Pérez-Valenzuela, Pablo Fuentealba, Alexies Dagnino-Subiabre

**Affiliations:** ^1^Laboratory of Stress Neurobiology, Center for Integrative Neurobiology and Pathophysiology, Institute of Physiology, Faculty of Sciences, Universidad de Valparaíso, Valparaíso, Chile; ^2^Department of Bioengineering, University of California, San Diego, La Jolla, CA, United States; ^3^Department of Psychiatry, Integrative Center for Neurosciences, Pontificia Universidad Católica de Chile, Santiago, Chile

**Keywords:** gamma oscillations, nucleus accumbens, stress, depression, social behavior

## Abstract

Alteration in social behavior is one of the most debilitating symptoms of major depression, a stress related mental illness. Social behavior is modulated by the reward system, and gamma oscillations in the nucleus accumbens (NAc) seem to be associated with reward processing. In this scenario, the role of gamma oscillations in depression remains unknown. We hypothesized that gamma oscillations in the rat NAc are sensitive to the effects of social distress. One group of male *Sprague-Dawley* rats were exposed to chronic social defeat stress (CSDS) while the other group was left undisturbed (control group). Afterward, a microelectrode array was implanted in the NAc of all animals. Local field potential (LFP) activity was acquired using a wireless recording system. Each implanted rat was placed in an open field chamber for a non-social interaction condition, followed by introducing another unfamiliar rat, creating a social interaction condition, where the implanted rat interacted freely and continuously with the unfamiliar conspecific in a natural-like manner (see Supplementary Videos). We found that the high-gamma band power in the NAc of non-stressed rats was higher during the social interaction compared to a non-social interaction condition. Conversely, we did not find significant differences at this level in the stressed rats when comparing the social interaction- and non-social interaction condition. These findings suggest that high-gamma oscillations in the NAc are involved in social behavior. Furthermore, alterations at this level could be an electrophysiological signature of the effect of chronic social stress on reward processing.

## Introduction

Humans as well as many other mammalian species exhibit social behaviors which imply several evolutionary advantages (Alexander, 1974). Moreover, social reward is crucial for emotional well-being; therefore, and impairment in this domain, is a key symptom in mood disorders (American Psychiatric Association, 2013). One of these conditions is depression; this disorder is characterized by “anhedonia” –the decreased reactivity to pleasurable stimuli – as well as by a deficiency in reward processing (Admon and Pizzagalli, 2015).

It has been shown that the NAc, that is part of the ventral striatum, is involved in motivation to carry out social interactions (Dolen et al., 2013). Additionally, reward conditioning for both drug and social interaction, leads to an increase of the electrical network activity in the NAc (Kummer et al., 2015). Likewise, clinical studies suggest that the NAc reward responsivity is altered in depressive patients (Pizzagalli et al., 2009; Misaki et al., 2016), and there is also evidence about the antidepressive effect of targeting the NAc with deep brain stimulation in patients suffering treatment-resistant depression (Schlaepfer et al., 2008; Nauczyciel et al., 2013).

Neural oscillations at the EEG or LFP levels, have been correlated to the activity of several cognitive functions in cortical and sub-cortical structures (Bosman et al., 2014), and they have also been linked to the symptomatology of neuropsychiatric disorders (Herrmann and Demiralp, 2005; Uhlhaas and Singer, 2010). Studies carried out in humans show that alterations among gamma oscillations, in EGG (30–100 Hz), represent an element of major depression (Herrmann and Demiralp, 2005; Uhlhaas and Singer, 2010). In line with this, gamma oscillations in the NAc of humans (Cohen et al., 2009; Lega et al., 2011) and rats (Berke, 2009; van der Meer and Redish, 2009) are evoked during reward processing. However, the role of gamma oscillations in the NAc during social interaction in healthy and depressive subjects remains unknown.

Negative stress or distress is a key environmental risk factor for mood disorders, such as major depression (Monroe et al., 2014; Pizzagalli, 2014). Distress can give way to depressive symptoms in healthy individuals (Berenbaum and Connelly, 1993; Charney and Manji, 2004), as well as deficit in brain reward system functions in laboratory animals (Der-Avakian et al., 2014; Donahue et al., 2014). Distress has also been shown to disrupt reward learning in humans (Bogdan and Pizzagalli, 2006; Pizzagalli et al., 2007; Bogdan et al., 2010, 2011) and rats (Der-Avakian et al., 2017). In line with this, CSDS experiment is carried out with an animal model which is commonly used to study susceptibility to depressive-like behaviors (Hammels et al., 2015). CSDS strongly decreases the reward system activity resulting in an long lasting anhedonic response in stressed rats (Der-Avakian et al., 2014), an element which can be reverted by an antidepressant treatment in mice (Berton et al., 2006; Tsankova et al., 2006; Krishnan et al., 2007). Several functional alterations in the NAc are induced by CSDS, not only at a molecular level, but also in neural-morphology and synaptic plasticity in rodents (Christoffel et al., 2011; Francis et al., 2015). Alterations in the metabolic profile and MSNs activity have been discovered in awake mice (Larrieu et al., 2017; Hamilton et al., 2018; Muir et al., 2018). The aforementioned evidence raises the question of what effects CSDS has on neural oscillations in the NAc when animals interact with a conspecific. In line with this, we hypothesized that CSDS disrupts gamma oscillations in the rat NAc during social interaction. In order to test this hypothesis, we performed *in vivo* electrophysiological recording in the NAc of stressed and non-stressed rats during social interaction with a conspecific. We found that gamma-band power in the NAc was higher in non-stressed rats during social interaction compared with the non-social interaction condition. Interestingly, gamma oscillations in stressed rats did not vary between the social and the non-social interaction conditions. These findings suggest that gamma oscillations are involved in social behavior, and that alterations at this level could be an electrophysiological signature of the effect of chronic social stress in reward processing.

## Materials and Methods

### Animals

Male *Sprague-Dawley* rats (380–420 g, 80–85 days old at the start of the experiment), commercially procured (Charles River Laboratories, Wilmington, United States), were used for the electrophysiological experiments and adult male Long Evans rats (700–850 g) commercially procured (Charles River Laboratories, Senneville, QC, Canada) were used as aggressors in the CSDS paradigm. All rats were maintained under a 12-h light–dark cycle (lights on at 8:00 am) and provided with food (Prolab RMH 3000, LabDiet^®^, MO, United States) and water *ad libitum*. Experiments were performed during the light phase. Animals were maintained in a temperature and humidity-controlled room (21 ± 1°C, 55%), and housed in groups of three before the electrode’s implantation. After surgery, and for the rest of the experiments, they were housed individually. Animals under the stress protocol were separated from non-stressed rats and kept in a different room specially designated for them. Body weights were monitored three times per week. All animal maintenance and experimentation procedures were approved by the Institutional Animal Ethics Committee of the Faculty of Sciences of the Universidad de Valparaíso (Chile) and were in strict accordance with animal care standards outlined in National Institutes of Health (United States) guidelines. Efforts were made to minimize the number of animals used and their suffering.

### Experimental Design

**FIGURE 1:**
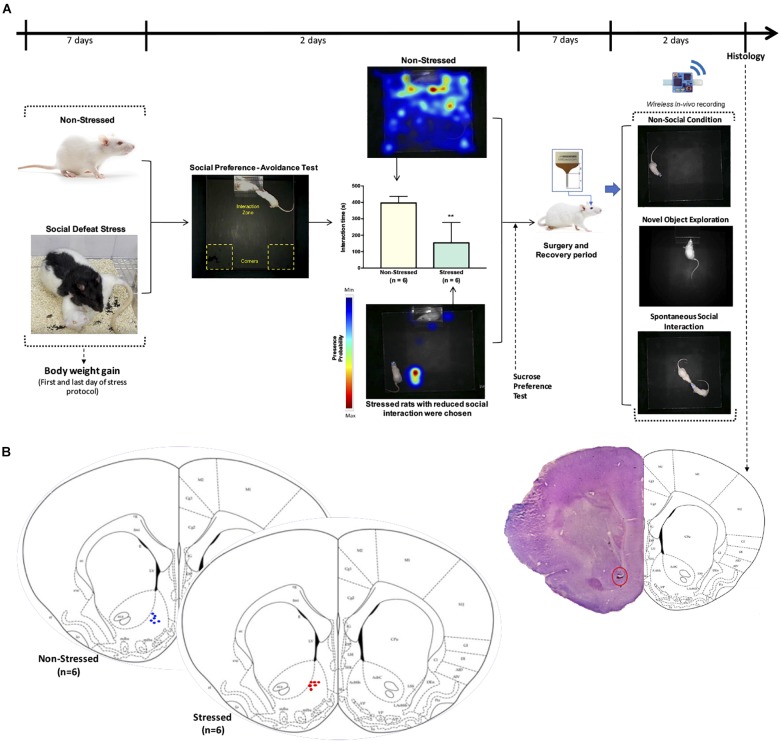
**(A)** Timeline of the experimental design. Male *Sprague-Dawley* rats were exposed to CSDS and a non-stressed group (*n* = 6) was left undisturbed. Afterward, non-stressed and defeated rats were subjected to open field test, social preference-avoidance test, and sucrose preference test. Stressed animals that had interaction times two standard deviation below the mean of non-stressed rats were chosen for stressed group (*n* = 6). Representative heat maps are shown for non-stressed and stressed rat’s tracking. A microelectrode array was implanted in the nucleus accumbens of all rats and local field potential activity was acquired using a wireless recording system in a non-social condition (open field), during exploration of a novel object, as well as when the implanted rats interacted freely and continuously with an unfamiliar conspecific in a natural-like manner. **(B)** Verification of electrode location. Schematic of final electrode locations in all rats. Each dot represents the electrode tips of one rat (*n* = 6 non-stressed and *n* = 6 stressed). Top right: example of a stained brain section with electrolytic lesions at the electrode tips (red circle).

Figure 1A shows the timeline of the experimental design. Body weight gain, social interaction, and sucrose preference were determined in both non-stressed rats and animals that were exposed to CSDS. Rats that were susceptible to CSDS had less social interaction as well as a decrease in sucrose preference in comparison with non-stressed rats. Three rats that were resilient to social stress were excluded from this study and used in another research. In the *in vivo* electrophysiological experiments, non-stressed rats (*n* = 6) were only compared with stress susceptible rats (*n* = 6; stressed group).

### CSDS Protocol

The CSDS protocol used in this study was modified from the resident-intruder model (Miczek, 1979). Animals from the stressed group were subjected to CSDS for seven consecutive days. During each episode of social stress, a *Sprague-Dawley* rat (intruder) was placed into the home cage of an unfamiliar male *Long-Evans* rat (resident). To classify the level of aggression before applying the CSDS protocol, the *Long-Evans* rats (resident) were previously exposed to five other adult male *Sprague-Dawley* rats (intruders) using the same resident-intruder paradigm described here. The resident rats who attacked at least 80% of the intruder rats in the first 60 s of the test were classified as highly aggressive. A typical agonistic encounter resulted in intruder subordination or defeat, signaled by the intruder assuming a supine position for approximately 3 s. After defeat, a wire mesh enclosure was placed into the cage to prevent physical contact between the resident and intruder, but allowing visual, auditory, and olfactory contact for the remainder of the 30-min defeat session. Rats were returned to their home cage after each session, and body weight gain was monitored daily to be used as a stress marker.

### Open Field Test

Locomotor activity and anxiety like-behavior were evaluated using the open field test. All animals were naive to the test. Rats were placed individually in the center of a black Plexiglass cage (70 × 70 × 40 cm) for 5 min.

Total distance travelled, speed mean, and the time that rats spent in the perimeter and center (anxiety-like behavior marker) were automatically analyzed from video recordings using EthoVision^®^ XT (Noldus, Wageningen, Netherlands).

### Social Preference-Avoidance Test

Rats were tested for social behavior using a social interaction paradigm (Francis et al., 2015; Zoicas and Neumann, 2016). The rats were placed in an open field (85 cm × 35 cm × 50 cm) of which contained a transparent perforated chamber (25 cm × 15 cm) containing an empty enclosure and located in a designated interaction zone; the aforementioned transparent perforated chamber, they explored for 2.5 min. Time spent in the interaction zone for the experimental rat was measured using EthoVision^®^ XT (Noldus, Wageningen, Netherlands). Afterward, the experimental rat explored the same perforated chamber for an additional 15 min after the introduction of a novel rat (male *Sprague-Dawley* of similar age) into the perforated chamber. The interactions were measured by the amount of time the experimental rat spent interacting with the other rat (considered as a stimulus). Representative heatmap for each experimental group were made using EthoVision^®^ XT. The experimenter was blind to experimental group conditions. Stressed rats that had interaction times two standard deviations below the mean of non-stressed rat group were chosen for our study.

### Sucrose Preference Test

As an efficacy measure for the CSDS protocol, depressive like-behavior was evaluated using the Sucrose Preference Test which evaluates the inability to experience pleasure in animals. The rats were first trained for 3 days to consume sweet liquid (5% sucrose), and water deprived for 12 h before the test. During the test, the rats were allowed to choose between two bottles for 1 h, one containing only water and the other containing a 5% sucrose solution. The amount of liquid consumed by the rats was measured, and the percentage of preference of sweet liquid in relation to the neutral liquid was calculated.

### Surgery

The pre-operatory protocol consisted of 10 mg/kg of doxycycline applied orally a day before the surgery, as well as acepromazine 2.5 mg/kg and atropine 0.1 ml (both intramuscular) 30 min before the surgery. Through the surgery, 5 mg/kg of subcutaneous ketoprofen, 10 mg/kg of subcutaneous doxycycline, and 10 mg/kg of intraperitoneal tramadol were administered. For the post-operatory period, 2.5 mg/kg of ketoprofen and 10 mg/kg of oral doxycycline were applied orally for 3 days.

Rats were anaesthetized with isoflurane (induction 3%, maintenance 2%) in O_2_. Body temperature was maintained at 37°C with a temperature controller system (RWD, Cat. No. 69001, Shenzhen, China). Rats were secured in a stereotaxic frame and unilaterally implanted in the left NAc with a microelectrode array aimed at the following coordinates: 1.8 mm anterior to the bregma, 1.0 mm lateral to the midline and 7.5 ventral from the skull (Paxinos and Watson, 2007). All implants were secured using dental cement. Rats were chronically implanted with a microelectrode array (Microprobes for Life Sciences, Gaithersburg, MD, United States) consisting of four individually insulated platinum/iridium wires (75 μm diameter, 3 MΩ, 250 μm distance between electrodes and rows) and attached to an 18-pin connector (Omnetics Connector Corp., Minneapolis, MN, United States). A local reference of the same metal, but lower impedance (10 KΩ) than the recording electrodes, was used. The ground consisted of a stainless-steel wire connected to the skull via a screw positioned on the cerebellum area.

After surgery, rats were allowed to recover for 7 days. They were habituated daily, including handling and head manipulation to avoid any possible stress that could be generated by the head-stage connection during the recordings.

### *In vivo* Electrophysiology

The data were acquired with a wireless system (W2100, Multichannel Systems MCS GmbH, Harvard Bioscience, Inc., Reutlingen, Germany) using a 16-channels headstage with an amplifier bandwidth at 1 Hz to 5 kHz and a sampling rate at 25 kHz (gain at 101, input impedance at 1 GΩ, resolution of 16 bit, input voltage range of ±12.4 mV, input noise of <1.9 μVRMS and the distance for wireless link of 5 m). The acquisition software was the Multichannel Suite (Multi Channel Systems MCS GmbH, Harvard Bioscience, Inc., Reutlingen, Germany).

The signals were down-sampled offline to 1 kHz and bandpass filtered between 30 and 100 Hz. Electrodes with poor signal quality and movement artifacts were visually rejected by two researchers using EEGLab (Delorme and Makeig, 2004), based on spectral and time-domain characteristics, and confirmed by automated movement analysis of the recorded videos.

Spectral analysis was performed with the multitaper method using the Chronux toolbox (Bokil et al., 2010). Not all recordings had the same length because the headstage in some rats was disconnected before ending the testing procedure. So to guarantee comparability between animals, only the first 150 s of signal were extracted at each condition and multitaper parameters were set using window lengths of *T* = 2 s with 1.9 s overlap; time-bandwidth product TW = 2, and number of tapers *K* = 3. The median power at the 30–60 Hz and 61–90 Hz frequency range was calculated for each condition, and the linear scale changed to dB for the spectra plotting and statistical analysis.

### Sample Size for *in vivo* Recording Experiments

The number of implanted rats that were used in our study was comparable with previously published studies on stressed rats (Jacinto et al., 2013, 2016). Thus, fifteen rats were implanted in the NAc (non-stressed, *n* = 8; stressed group, *n* = 7), three rats were discarded from the experiments, because one rat did not undergo the recording procedure due to a premature detachment of the implant, and two rats were discarded because of microelectrode failure. Finally, twelve implanted rats were used in the electrophysiology experiments (non-stressed, *n* = 6; stressed group, *n* = 6).

### Behavioral Testing

Prior to the behavioral testing, rats were habituated to the testing room for 30 min, 3 days consecutively. Non-stressed and stressed rats were subjected to a test consisting of three phases: in the first one, they were introduced for 5 min in an open field consisting of a square Plexiglass cage (70 × 70 × 40 cm), in which animals could freely explore (non-social condition). Then, a novel object consisting of a small cube of transparent acrylic was introduced into the box, allowing them to freely explore it for 5 min, followed by the removal of the novel object. Finally, another *Sprague-Dawley* rat of the same sex, with similar weight and previously evaluated as “non-aggressive” was introduced into the open field, allowing the animals to freely interact in a natural-like manner (social condition) for 5 min (see Supplementary Videos S1, S2). For the subsequent analysis of the behaviors, all tests were recorded with a video system, integrated, and synchronized with the electrophysiological recording (W2100-Video-System, Multichannel Systems MCS GmbH, Harvard Bioscience, Inc., Reutlingen, Germany). In addition, LFP acquisition was also synchronized with video recordings of the animal tracking and automatized analysis with EthoVision^®^ XT (Noldus, Wageningen, Netherlands) to measure the speed and space-coordinates of the animals when they were performing the tasks.

### Histology

After data collection was complete, a 25 μA current was passed through the electrodes for 20 s each. Three days following gliosis, rats were anesthetized with isoflurane and perfused intracardially with 4% paraformaldehyde in PBS. Brains were extracted and stored in 4% paraformaldehyde with 30% sucrose before being cut in 50-μm sections using a cryostat microtome (Kedee KD-2950, Zhejiang Jinhua Kedi Instrumental Equipment Co., Ltd., Zhejiang, China). Sections were mounted on gelatin-coated slides and stained using the Nissl method for localization of recording locations. Only data from electrodes with confirmed recording locations in the NAc were analyzed (Figure 1B).

### Statistical Analyses

Data was analyzed using Prism 7 (GraphPad Software Inc., La Jolla, CA, United States), IBM SPSS^®^ (IBM Corp, New York, NY, United States), or MATLAB (MathWorks, Natick, MA, United States). All variables that met the normal distribution test, using the Shapiro–Wilk test, and homoscedasticity, using the Levene test, were analyzed with parametric statistics. When the criteria for normality and homoscedasticity were not met, the data were analyzed with nonparametric statistics (Mann Whitney test or Wilcoxon test).

We used a two-tailed unpaired *t* test to compare the non-stressed and stressed groups in the behavioral parameters of the open field test, social interaction test, the sucrose preference test, and for body weight gain.

For the analysis of locomotor activity, we identified the events when the animal showed a speed, two standard deviations above (“fast movement”) vs. below (“slow movement”) the mean of locomotion, followed by the use of a two-tailed paired *t* test to compare the mean power for fast and slow movement events across the low and high-gamma frequency bands.

The Wilcoxon test was used for within a group of animals for comparison in regards to the power of low- and high-gamma oscillations in the non-social, novel object exploration, and social condition. Mann Whitney test was used to determine how significant was the percentage of change was between the non-social and social condition for the non-stressed and stressed groups in low (30–60 Hz) and high-gamma bands (61–90 Hz).

A probability level of 0.05 or less was accepted as significant. Results were expressed as the median, the 95% confidence interval of the spectral power (spectral plots) and as the median percentage of change in gamma-power during social interaction with respect to the non-social condition (open field exploration, considered as basal) with its minimum and maximum values (whiskers) in the box-and-whiskers plots.

## Results

### Effects of CSDS on Weight Gain, Social Interaction, and Depressive-Like Behavior

**FIGURE 2:**
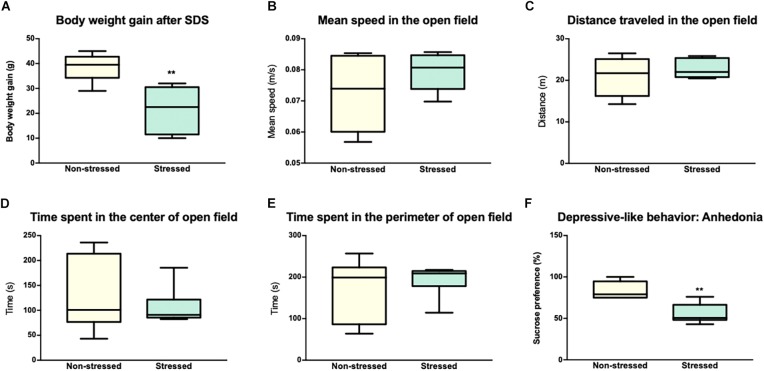
Socially defeated rats showed an alteration in body weight gain, depressive like-behavior, and a normal performance in the open field. **(A)** Animals subjected to chronic social defeat stress (CSDS) showed a decrease in body weight gain compared to animals in the non-stressed group. The graph shows the body weight gain (grams) of the rats after 7 days of stress or 7 days without treatment in the case of non-stressed. **(B,C)** Locomotor activity in the open field. Distance traveled and average speed of the animals in the open field were similar in both groups. **(D,E)** Evaluation of anxiety-like behavior in the Open Field Test (time in the center and time in the perimeter). No statistically significant differences were observed between the groups in any of the parameters studied in the Open Field. **(F)** Evaluation of depressive-like behavior in the sucrose preference test. The animals of the stress group showed a significant decrease in the preference of sucrose compared to non-stressed rats. The values are presented as the median with its minimum and maximum. ^∗∗^*p* < 0.001.

Figure 1A shows that social interaction in the social preference-avoidance test was impaired in the rats that were exposed to CSDS (stressed group = 153.3 ± 124.5 s; non-stressed group = 395.8 ± 40.5 s, *P* = 0.0011). Maximum value of social interaction time (309.8 s) for stressed rats was two standard deviations below the mean of the non-stressed group (395.8 ± 40.5 s). In addition, stressed rats gained less weight than non-stressed rats (*t* = 3.917, df = 10; *P* = 0.0014) (Figure 2A). Along with this, the locomotor activity and anxiety-like behaviors of the animals were evaluated in the open field test (Figure 2B–E). Distance traveled and average speed were similar in all groups (*t* = 1.141, df = 10; *P* = 0.2804 and *t* = 0.8302, df = 10; *P* = 0.4258, respectively). Regarding anxiety-like behaviors, no significant differences were observed in the time that animals spent in the center (*t* = 0.6661, df = 10; *P* = 0.5204) or in the perimeter (*t* = 0.6661, df = 10; *P* = 0.5204) of the open field. Additionally, the *t* test showed differences between the groups in depressive-like behaviors based on the sucrose preference test (*t* = 4.277, df = 10; *P* = 0.0016), which allows obtaining an indicator of anhedonia in the stressed rats (Figure 2F).

### Neural Oscillations in the NAc and Sociability of the Rats

**FIGURE 3:**
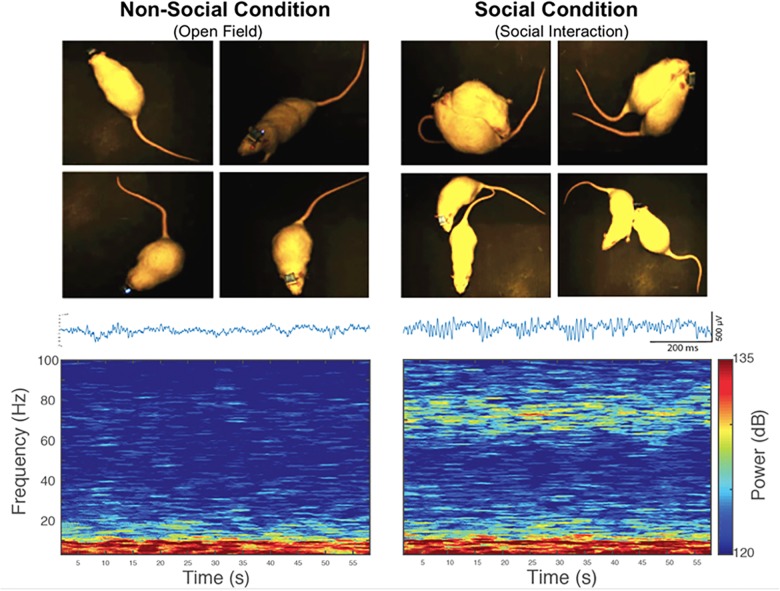
An increase in the power of high-gamma oscillations in the rat nucleus accumbens during spontaneous social interaction in comparison to a non-social condition. Left: Representative picture of a rat exploring an open field (non-social condition) and then interacting socially with a conspecific (right). Bottom left: representative traces of LFP signal and spectrogram of the LFP registered in the NAc of a rat during 60 s of exploratory behavior in an open field (non-social condition). Bottom right: representative traces of LFP signal and 60-s spectrogram of spontaneous social interaction recorded in the same rat. Gamma oscillatory activity is prominent during spontaneous social interaction compared to the non-social condition.

Our first electrophysiology experiment investigated a possible relationship between LFP oscillations in the NAc and social interaction. With this purpose in mind, we implanted 4-channels microelectrode arrays into the NAc of the six non-stressed rats from the previous behavioral measures (Figure 1A). Then, we recorded *in vivo* LFP activity in the NAc when implanted rats performed spontaneous social interactions with a novel conspecific, using a wireless recording system. In this test, the rats were continuously interacting for 5 min (Figure 1A, see section “Materials and Methods”). For the analysis, the first 150 s of signal was extracted at each condition. We found that when rats experienced social interactions, gamma-band power in the NAc was higher in the high-gamma frequency band (61–90 Hz) compared to the non-social condition (*P* = 0.010) (see Supplementary Figure S1). In low-gamma, on the other hand, no significant differences were observed between both conditions (*P* = 0.094) (Figure 3, 6A,C).

**FIGURE 4:**
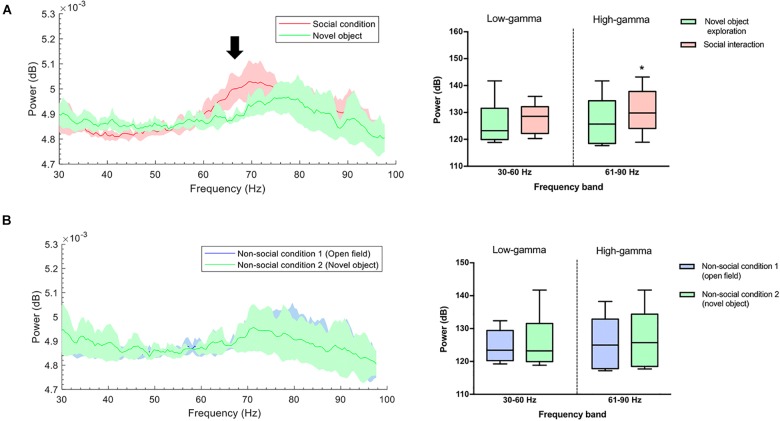
**(A)** Gamma-band power during novel object recognition was lower compared with a social condition. Left: Group-median power spectra (solid line, median; shaded area, 25th–75th percentile) across the 30–100 frequency band showing increased power of the high-gamma frequency band (black arrow) during spontaneous social interaction (red) in comparison to the non-social condition/novel object (green). Right: Mean power (dB) for both conditions across two gamma frequency bands (low-gamma: 30–60 Hz and high-gamma: 61–90 Hz). The whiskers indicate the minimum and maximum values. ^∗^*p* < 0.05. **(B)** The power of gamma oscillations between two non-social conditions was similar. Left: Group-median power spectra (solid line, median; shaded area, 25th–75th percentile) across the 30–100 frequency showing no differences between non-social condition 1 (open field) and non-social condition 2 (novel object) (green and blue line, respectively). Right: Mean power (dB) for both conditions across two gamma frequency bands (low-gamma: 30–60 Hz and high-gamma: 61–90 Hz). The whiskers indicate the minimum and maximum values.

To exclude the effect of novelty as a confounding factor, the animals were subjected to a second non-social condition, which consisted of exploring a novel object. A significant difference was found in gamma oscillations between the social interaction and the exploration of the new object in the 61–90 Hz frequency band (*P* = 0.031) (Figure 4A), and not in the 30–60 Hz band (*P* = 0.313). The animals in both non-social conditions showed similar gamma-power at frequencies 30–60 Hz (*P* = 0.563) and 61–90 Hz (*P* = 0.063) (Figure 4B).

**FIGURE 5:**
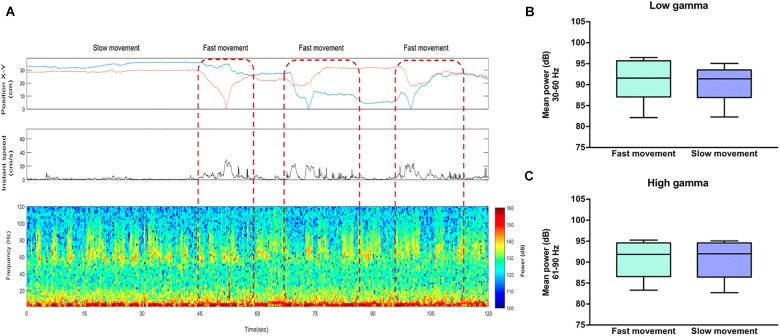
High-gamma oscillations in the rat nucleus accumbens are independent of locomotor activity. **(A)** Top: The first box represents the position of the rat in a square arena and the second box represents the instant speed. Bottom: Representative LFP spectrogram from the NAc of a rat socially interacting. Red dotted lines indicate the events when the animal showed a speed two standard deviations above (“fast movement”) vs. below (“slow movement”) the mean of locomotion. **(B)** Mean power (dB) for fast and slow movement events across the low-gamma (30–60 Hz) and **(C)** high-gamma (61–90) frequency bands. The whiskers indicate the minimum and maximum values. The *t*-test shows no significant difference in low and high-gamma power between fast and slow movement.

Subsequently, to evaluate whether this increase in high-gamma power was the mere consequence of motor related behavior, we analyzed those events offline when the animal increased the level of movement during social interaction (“fast movement”) vs. those events when the animal moved slowly (“slow movement”) using synchronized video recordings of the animal tracking and an automatized analysis with EthoVision^®^ XT (Noldus, Wageningen, Netherlands) to measure the speed and space-coordinates of the animals when they were performing the tasks. We observed that the power of gamma oscillations was independent of the locomotor activity during social interaction both in the 30–60 Hz band (*t* = 1.007, df = 5; *P* = 0.3602) and in the 61–90 Hz (*t* = 0.0268, df = 5; *P* = 0.9791) (Figure 5).

### Effects of CSDS on High-Gamma Oscillations in the NAc

**FIGURE 6:**
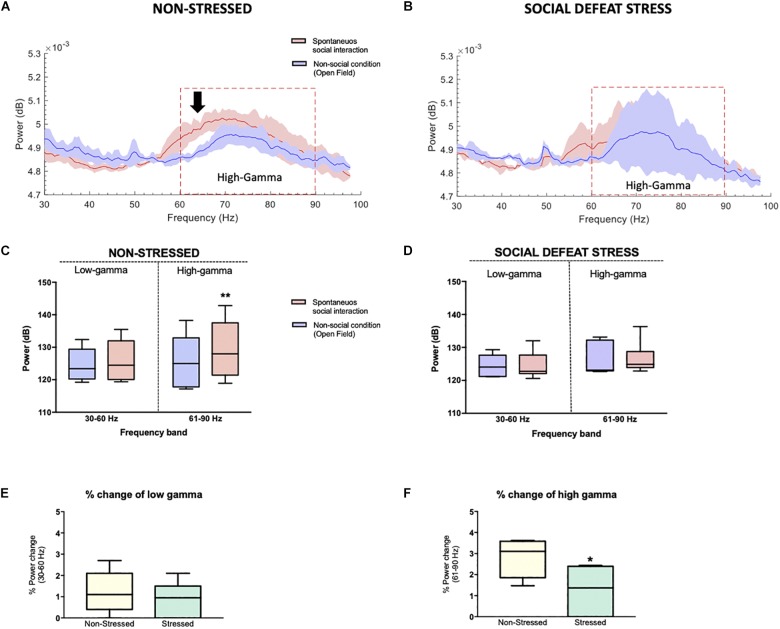
Stressed rats did not show differences in the power of high-gamma oscillations in the NAc between the social and non-social condition: **(A)** Non-stressed group-median power spectra (solid line, median; shaded area, 25th–75th percentile) across the 30–100 frequency band showing increased power of the high-gamma frequency band (black arrow, red dotted line) during the spontaneous social interaction (red) in comparison to the non-social condition (blue). **(B)** Stressed group-median power spectra (solid line, median; shaded area, 25th–75th percentile) across the 30–100 frequency band showing no differences in the power of high-gamma (red dotted line) during spontaneous social interaction (red) in comparison to the non-social condition (blue). **(C,D)** Mean power (dB) for both conditions in two gamma frequency bands (30–60 Hz or “low-gamma,” 61–90 Hz or “high-gamma”) for non-stressed and stressed implanted rats. The whiskers indicate the minimum and maximum values. A significant difference was observed between the two conditions for the high-gamma frequency band. ^∗∗^*p* < 0.01. Median percentage of change in low **(E)** and high-gamma **(F)** power during spontaneous social interaction compared with the non-social condition (open field exploration, considered as basal) with its minimum and maximum values. Rats that were exposed to CSDS had a decrease of % power change in high-gamma band. ^∗^*P* < 0.05.

The electrophysiology study involved the six stressed rats from the previous behavioral measures, which were implanted in the NAc (Figure 1A). No significant differences were observed for non-social and social condition in the high-gamma (*P* = 0.438) or low-gamma (*P* = 0.438) frequency band for rats that were exposed to CSDS (Figure 6B,D).

The unpaired *t* test showed that the percentage of power change between the social and non-social condition was lower for the implanted rats of the stressed group in the high-gamma band in comparison to non-stressed rats (*P* = 0.041) while no differences were found in the low-gamma band (*P* = 0.738) (Figure 6E,F).

## Discussion

The innovative aspect of our research considered the use of technology that allowed us to integrate an assessment of social interaction based on a natural-like interaction with the *in vivo* recordings of LFP oscillations in the NAc of stressed rats. The main objective of this study was to find the electrophysiological correlate of social interaction in an essential area for reward processing such as the NAc. Although there was already some evidence about the participation of the NAc in social behaviors (Alkire et al., 2018; Warnell et al., 2018) to date, it was unknown that social interaction correlated with the increase in high-gamma power (61–90 Hz, in the present study) in the NAc, which points to a possible brain oscillatory modulation based on social interaction, in that specific region, in high-gamma (Figure 6). In contrast, high-gamma power in the NAc of stressed rats was independent of social interaction (Figure 6).

It should be noted that the origin of gamma oscillations in the NAc could theoretically be attributed to different factors, including synaptic inputs that come from afferent areas such as the prefrontal cortex, the hippocampus, the amygdala, the VTA, and the thalamus (Colgin et al., 2009; Popescu et al., 2009; Muir et al., 2018). Also, they may be attributed to intrinsic local membrane potentials and multiunit activity of spikes (van der Meer et al., 2010), or to the synaptic currents resulting from local projections within the NAc. Some authors refer to the NAc as a “switchboard” (Redgrave et al., 1999; Goto and Grace, 2008; Gruber et al., 2009). This proposal implies that NAc is more than a simple passive receiver of incoming signals from afferents; rather, the NAc has a mechanism for the exchange between signals. In such an exchange scenario, the NAc would be able to “select” a dominant input. This suggests, for example, that during high-gamma oscillatory activity in the NAc, other areas that also exhibit such activity, as the prefrontal cortex, could be functionally synchronized with the NAc in non-stressed rats, having an influence on their behavior; on the other hand, while during low-gamma the piriform cortex or the amygdala – which also exhibit oscillations in this frequency band – would be controlling the output of the NAc (van der Meer et al., 2010). However, it might be that the NAc has the role of signal discrimination, allowing the selection and exchange of certain signals, passing them from one source to the next. In this regard, it has been reported that distress is related to alterations in synaptic transmission and volume atrophy in cortical and limbic regions, such as the prefrontal cortex (Holmes and Wellman, 2009; Negron-Oyarzo et al., 2014) and the hippocampus (McEwen, 2016; Perez et al., 2018). The aforementioned could affect the functional connectivity between the cortex and NAc in stressed animals, inducing the decrease of gamma power that we found in the stressed rats (Figure 6B,D). This could lead to a failure in the integration of cortical information in the NAc (Mallet et al., 2005), possibly from prefrontal cortex inputs, a brain region involved in social behavior, according to recent evidence (Alkire et al., 2018).

Regarding the possible origin of the gamma oscillations within the NAc, a reciprocally connected inhibitory network at NAc could generate gamma activity, as was proposed by Buzsaki and Wang, 2012, with parvalbumin positive fast spiking interneurons (PV+ FSI) which inhibit the MSNs (Mallet et al., 2005). The NAc is a subdivision of the ventral striatum. Furthermore, the coupling to gamma rhythms has been described as being selective for the PV+ FSI in the stratium (Berke, 2009), which has been theoretically linked to the generation of oscillations in this frequency band (Popescu et al., 2009).

It is known that PV+ FSI strongly inhibit projection neurons in the NAc, which do not seem to inhibit each other significantly (Jaeger et al., 1994). Therefore, PV+ FSI are probably the main neurons responsible for the GABAergic inhibition of the striatum; this strongly limits the striatal output (Nisenbaum and Berger, 1992). Although PV+ FSI constitute only 1–2% of the neurons in the NAc, they provide a powerful feedforward inhibition that controls the firing of the MSNs (Warren and Whitaker, 2018). It becomes evident then that by exciting PV+ FSI and by reducing their synaptic inhibition, dopaminergic afferents can exert an important inhibitory influence in the striatum. Particularly, dopamine (DA) can critically regulate feedforward inhibition, which is a major feature of cortico-striatal communication (Koos and Tepper, 1999).

According to previous evidences, a possible explanation would be that under normal conditions, the increase of the neurotransmitter DA in the NAc during social interaction would excite the PV+ FSI directly through depolarization mediated by D1-type receptors, or indirectly, reducing its synaptic inhibition through presynaptic D2-type receptors (Bracci et al., 2002). This would increase the inhibitory activity of the PV+ FSI on the MSN, which would generate oscillations in gamma frequency. Therefore, in the case of stressed rats when interacting socially with another unfamiliar conspecific, it is proposed that there would be (1) a decrease in the levels of DA in the NAc compared to non-stressed rats in the same behavioral event, which leads to (2) a decrease in the activity of the PV+ FSI that generates (3) the alteration of the local inhibitory circuit between the PV+ FSI and the MSN and leads to (4) the decrease of gamma oscillations in the NAc (Buzsaki and Wang, 2012) during social interaction. Analyzing each aspect of this idea, the first element is the possible decrease in DA levels in the NAc of rats that were exposed to CSDS. Although this was not measured in the present study, there is previous evidences that supporting this idea and additional evidence that is contrary to the aforementioned. For example, an increase of activity of VTA DA neurons projecting to NAc and activation of D1-MSN is needed to initiate the social behavior (Gunaydin et al., 2014). In this regard, the study of Muir et al. (2018) is close to our findings because they measured D1-MSN activity specifically when implanted mice were carrying out social interaction. Authors found that social interaction evokes low levels of D1-MSN activity in pro-susceptible mice compared with pro-resilient mice (Muir et al., 2018). This evidence suggests that social interaction did not stimulate much DA release toward the NAc in susceptible mice, compared with resilient or non-stressed animals. In line with this, optostimulation of VTA DA in mice exposed to chronic mild stress decreases depressive-like behaviors (Tye et al., 2013), as well as chronic stress which leads to a decrease in dopamine levels in the NAc (Lehner et al., 2018). Conversely, phasic optostimulation of VTA DA neurons produce the susceptible behavioral phenotype in mice (Chaudhury et al., 2013) and susceptible mice show an increase in the firing rate of VTA DA neurons and D1-MSN hyperexcitability (Cao et al., 2010; Francis et al., 2019). This evidence suggests that the effects of stress on the VTA-NAc pathway depend on the type of stressor and its intensity. In this scenario, a fundamental piece in this puzzle is missing. DA concentration in the NAc has not yet been determined during social interaction in stressed rodents. It is possible that chronic exposure to CSDS could increase phasic firing rate of VTA DA neurons, which in turn desensitizes the D1-MSNs in the NAc and impairs social interaction.

At a clinical level, it has been shown that a rapid antidepressant effect of ketamine, an NMDA receptor antagonist, depends partly on the mesolimbic dopaminergic system (Belujon and Grace, 2014; Duman, 2018). In line with this, ketamine restores reduced VTA DA neuron activity of mice exposed to chronic mild stress (Rincón-Cortés and Grace, 2017). This evidence suggests that an antidepressant effect of ketamine could be induced by an increase of the VTA-NAc activity, which could be related with an increase in high gamma oscillations in the NAc during social behavior.

In conclusion, similar to previous studies that showed that gamma oscillations in the NAc are associated with the processing of natural rewards (e.g., food intake) or synthetic rewards such as drug use (Berke, 2009; Cohen et al., 2009; van der Meer and Redish, 2009; Lega et al., 2011), these results suggest, specifically, that high-gamma oscillations could also be involved in the rewarding effect generated by the presence of a conspecific, as well as socially interacting with it, and that chronic social stress disrupts these oscillations. Alterations in this domain seem to be involved in the etiology of neuropsychiatric disorders related to stress, such as major depression and anxiety disorders.

## Data Availability

The datasets for this manuscript are not publicly available because they have not been published yet. Requests to access the datasets should be directed to alexies.dagnino@uv.cl.

## Ethics Statement

All animal maintenance and experimentation procedures were approved by the Institutional Animal Ethics Committee of the Faculty of Sciences of the Universidad de Valparaíso (Chile) and were in strict accordance with animal care standards outlined in the National Institutes of Health (United States) guidelines. Efforts were made to minimize the number of animals used and their suffering.

## Author Contributions

AI-M and AD-S designed the research, analyzed the data, and wrote the manuscript. AI-M, MA-R, MA-S, CP-V, and PF performed the research. All authors contributed to the final version of the manuscript for submission.

## Conflict of Interest Statement

The authors declare that the research was conducted in the absence of any commercial or financial relationships that could be construed as a potential conflict of interest. The reviewer AD-A, declared a shared affiliation, with no collaboration, with one of the authors, MA-R, to the handling Editor at the time the of review.

## Abbreviations

CSDS: chronic social defeat stress
dB: decibels
EEG: electroencephalography
GABA: gamma aminobutyric acid
Hz: Hertz
LFP: local field potential
MSNs: medium spiny neurons
NAc: nucleus accumbens
PV+ FSI: fast spiking interneurons expressing parvalbumin
SSR: sequential stopping rule
VTA: ventral tegmental area
