# Supplementary figures and images for: Impact of Stress on Gamma Oscillations in the Rat Nucleus Accumbens During Spontaneous Social Interaction
Source: Front Behav Neurosci. 2019 Jul 10;13:151. doi: 10.3389/fnbeh.2019.00151 (PMC6636240; doi:10.3389/fnbeh.2019.00151)

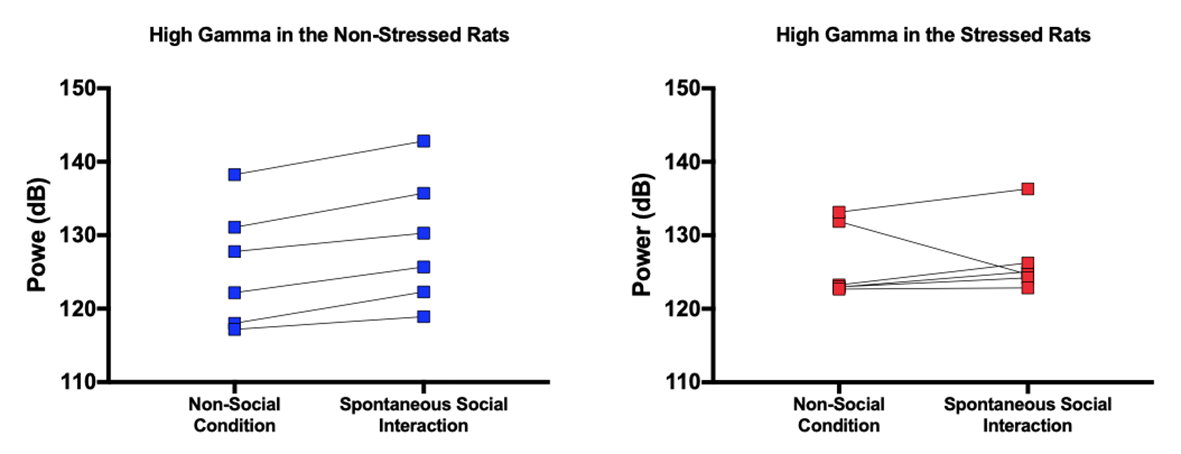

Supplement: Supplementary file 1 [file Image_1.TIF]
